# Treatment Approaches for Therapy-Associated Mucosal Lesions in Pediatric Oncohematology Patients: Findings from a Retrospective Observational Study in an Italian Pediatric Hospital

**DOI:** 10.3390/healthcare14162453

**Published:** 2026-08-08

**Authors:** Biagio Nicolosi, Emanuele Buccione, Hamilton Dollaku, Matilde Molini, Benedetta Virginia Difalco, Vincenzo Nobile, Giorgio Reggiardo, Daniele Ciofi, Annalisa Tondo, Greta Ghizzardi, Rosario Caruso, Guido Ciprandi

**Affiliations:** 1 Healthcare Professions Department, Meyer Children’s Hospital IRCCS, 50139 Florence, Italy; benedetta.difalco@meyer.it (B.V.D.); daniele.ciofi@meyer.it (D.C.); 2 AISLeC, Italian Nursing Association of Wound Care, 47923 Rimini, Italy; 3 Pediatric Intensive Care Unit, Local Health Authority 3 of Pescara, 65124 Pescara, Italy; emanuele.buccione@asl.pe.it; 4 Cardiac Rehabilitation Unit, IRCCS Don Gnocchi Hospital, 50124 Florence, Italy; hdollaku@dongnocchi.it; 5 Bachelor’s Degree Program in Nursing, School of Human Health Sciences, University of Florence, 50134 Florence, Italy; matilde.molini@stud.unifi.it; 6 R&D Department, Complife Italia S.r.l., 27100 Pavia, Italy; 7 Department of Biostatistics, Clinical Validation from Biopharmaceutical Findings (CVBF), 27100 Pavia, Italy; giorgioreggiardo@cvbf.net; 8 Department of Oncology, Hematology, Hematopoietic Stem Cell Transplantation (HSCT) and Gene Therapy, Meyer Children’s Hospital IRCCS, 50139 Florence, Italy; annalisa.tondo@meyer.it; 9 Directorate of Nursing and Allied Health Professions, ASST di Lodi, 26900 Lodi, Italy; greta.ghizzardi@asst-lodi.it; 10 Department of Biomedical Sciences for Health, University of Milan, 20133 Milan, Italy; rosario.caruso@unimi.it; 11 Health Professions Research and Evidence Transfer Unit, IRCCS MultiMedica, 20099 Sesto San Giovanni, Italy; 12 Complex Unit of Plastic Surgery, University of Padua, 35128 Padua, Italy; guido.ciprandi@unipd.it

**Keywords:** pediatric oncohematology, oral mucositis, oral stomatitis, perianal fissures, mucosal lesions, supportive care, healing time, nursing care

## Abstract

**Highlights:**

**What are the main findings?**

- Mucosamin^®^ Spray was associated with faster healing of oral stomatitis than conventional treatment.
- Mucosamin^®^ Rectal Gel was associated with faster healing of perianal fissures.

**What are the implications of the main findings?**

- The results support early, site-specific mucosal assessment and treatment in pediatric supportive care, suggesting that Mucosamin^®^-based approaches may help reduce lesion duration.
- Because the study was retrospective and observational, the findings should be considered preliminary and confirmed in prospective controlled multicenter studies.

**Abstract:**

**Background/Objectives**: To describe therapy-associated oral stomatitis and perianal mucosal lesions in pediatric oncohematology patients and compare healing time according to the treatment approach used in routine clinical practice. **Methods**: This retrospective observational study was conducted at Meyer Children’s Hospital, Florence, Italy. Electronic health records of patients admitted to the Oncology or Bone Marrow Transplantation units between 1 January and 31 December 2024, were screened. Eligible patients were aged 0 to 18 years and had documented oral stomatitis and/or perianal mucosal lesions with sufficient clinical and nursing documentation to determine lesion localization, treatment, clinical evolution, and healing time. Demographic, clinical, pharmacological, nutritional, and nursing variables were extracted. Oral mucositis severity was classified using the World Health Organization Oral Toxicity Scale. **Results**: Among 525 screened records, 89 eligible patients aged 5 months to 18 years were included in the final analysis. Lesions were distributed across three mutually exclusive categories: oral-only (39.3%), perianal-only (42.7%), and combined oral–perianal involvement (18.0%). Most oral cases were grade 2 to 4. Oral stomatitis treated with Mucosamin^®^ Spray healed faster than conventional therapy (3.5 ± 0.8 vs. 6.7 ± 1.1 days; mean difference, −3.21 days; 95% CI, −3.75 to −2.67; *p* < 0.001). Perianal fissures treated with Mucosamin^®^ Rectal Gel also showed a shorter healing time than conventional therapy (3.4 ± 0.8 vs. 6.1 ± 0.8 days; mean difference, −2.76 days; 95% CI, −3.30 to −2.21; *p* < 0.001). **Conclusions**: Mucosamin^®^ Spray and Rectal Gel were associated with shorter documented healing times than conventional approaches in this real-world pediatric cohort. These findings should be interpreted as preliminary and confirmed in controlled prospective multicenter studies.

## 1. Introduction

Oral mucositis is an inflammatory and ulcerative complication of the oral mucosa that commonly occurs as a consequence of anticancer treatments, including chemotherapy (CH), radiotherapy (RT), and conditioning regimens for hematopoietic stem cell transplantation [1]. It occurs in the pharyngeal, laryngeal, esophageal regions of the oral cavity and other areas of the gastrointestinal tract [2]. Typically, oral mucositis develops within 3 to 14 days after chemotherapy and may present with erythema, edema, ulceration, spontaneous bleeding, oral pain, pseudomembrane formation, dysphagia, and odynophagia [3,4].

Clinically, oral mucositis can substantially impair feeding, hydration, oral hygiene, communication, and treatment tolerance, particularly when lesions are painful or ulcerative [3,5,6,7]. Dysphagia and odynophagia may limit nutrient intake, leading to dehydration and weight loss and, in some cases, nutritional support with replacement or supple- mentation of feeding by mouth [3,8]. Other complications include infection risk, hospitalization, reduced quality of life, and the continuity of cancer treatment [9,10]. According to Villa et al., severe oral mucositis has been associated with dose modifications, treatment delays, or treatment interruptions in approximately 35% of patients, with potential consequences for therapeutic outcomes, quality of life, and healthcare costs [11].

The prevalence of oral mucositis is about 30 to 40% in the general adult cancer patient population, with higher incidence in patients receiving high-dose chemotherapy regimens or radiation [12,13]. In the pediatric population, the prevalence of oral mucositis is higher than that in the adult population, ranging from 40 to 90% [14,15,16].

In the pediatric population, oral mucositis is particularly relevant because its symptoms can be difficult to assess, especially in infants and younger patients who may be unable to describe pain or oral discomfort. In this context, oral mucositis may initially manifest as irritability, crying, refusal to eat or drink, excessive salivation, sleep disturbance, or reduced cooperation with oral care. Pediatric patients may also present with age-specific challenges related to oral examination, adherence to preventive measures, tolerance of topical products, and the feasibility of interventions such as oral cryotherapy or photobiomodulation therapy. This challenge is compounded by the limited availability of instruments for measuring mucositis in children that have been appropriately developed, validated, or evaluated for pediatric use [17,18]. These aspects make pediatric oral mucositis a complex supportive-care issue requiring age-adapted assessment and multidisciplinary management. In pediatric oncohematology patients, mucosal injury may also involve non-oral sites of the gastrointestinal tract, including the perianal region. For this reason, the present study considered oral stomatitis and perianal fissures as distinct clinical manifestations of therapy-associated mucosal injury, while analyzing them separately according to lesion localization and treatment approach. Given its clinical impact and the vulnerability of pediatric patients, oral mucositis should be regarded as a priority area in pediatric supportive care, requiring early recognition, standardized assessment, and individualized prevention and management strategies [16].

Several interventions have been proposed for the prevention and management of oral mucositis, including oral hygiene protocols, analgesic strategies, anti-inflammatory agents, antimicrobial approaches, cryotherapy, photobiomodulation, and barrier-forming or mucosal-protective products [19,20]. Nevertheless, despite these advances, oral mucositis remains a challenging and clinically relevant complication, particularly in pediatric patients. In this population, the available evidence on the efficacy and safety of preventive and therapeutic interventions is still limited, and not all treatment options used in adults are suitable or feasible for children because of age-related, clinical, and tolerability considerations [1,21]. Additionally, most of the studies have been performed on adults [1,22].

Current therapeutic options may reduce the incidence, severity, duration, or symptoms of oral mucositis in selected clinical settings; however, none can be considered universally effective for complete prevention or for ensuring rapid and complete resolution once lesions have developed. This limitation is particularly relevant in pediatric patients, where evidence remains heterogeneous, sometimes conflicting, and insufficient to establish standardized protocols for all interventions [1,16,23]. Therefore, there remains a significant unmet need for safe, well-tolerated, age-appropriate, and clinically effective strategies capable of both preventing oral mucositis and supporting mucosal healing [24,25].

This retrospective observational study aimed to describe the clinical characteristics of therapy-associated mucosal lesions in pediatric oncohematology patients and to compare healing time according to the treatment approach used in routine clinical practice.

## 2. Materials and Methods

This retrospective observational study was conducted at Meyer University Hospital (Azienda Ospedaliero-Universitaria Meyer), Florence, Italy. The hospital includes a dedicated pediatric oncohematology unit and manages more than 170 pediatric cancer cases per year.

Among the 525 electronic health records screened, 107 patients had documented oral stomatitis and/or perianal mucosal lesions. Eighteen patients were excluded because the available clinical or nursing documentation was insufficient to reliably determine one or more variables required for the analysis, including lesion localization, treatment approach, treatment initiation, clinical evolution, and healing time. Therefore, 89 patients were included in the final analysis.

### 2.1. Data Collection Procedures

Data were retrospectively collected by a trained researcher between May and July 2025. Data extraction was performed using available clinical and nursing records, with the aim of obtaining information relevant to the characterization of pediatric patients with oral mucositis and to the evaluation of the clinical course following treatment.

The collected data included demographic, clinical, pharmacological, and nursing-related variables, as follows:

- Demographic characteristics comprised age at the time of observation, sex, weight and cancer type;
- Clinical data included the severity of oral stomatitis and perianal lesion presence. Severity of oral stomatitis was classified according to the World Health Organization (WHO) Oral Toxicity Scale [13]. The WHO classification was applied only to patients with documented oral involvement. Perianal lesions were recorded separately according to their documented presence, localization, and clinical course, as the WHO Oral Toxicity Scale is specific to oral mucosal toxicity and is not applicable to extraoral lesions.
- Nursing data included the specific topical treatment used for oral stomatitis or perianal mucosal lesions. Patients were classified according to the principal topical treatment documented in the medical record. The conventional treatment group comprised predefined topical regimens routinely used in clinical practice, including honey alone or vitamin E spray-based combinations with chlorhexidine, nystatin, or honey. These regimens were considered alternative treatment strategies rather than concomitant topical therapies. Accordingly, each patient was assigned to a single treatment group based on the principal therapeutic approach received. Healing time was defined as the number of days between treatment initiation and the first clinical documentation of lesion resolution. When complete resolution was not explicitly documented, the first record indicating clear clinical improvement was not considered as evidence of complete healing and was therefore excluded from the healing time analysis.

All collected variables were reviewed and analyzed to describe the study population, characterize the severity and clinical context of oral mucositis, and evaluate treatment- related outcomes in terms of mucosal healing time.

### 2.2. Eligibility Criteria

Patients were eligible for inclusion if they met all the following criteria: (1) age between 0 and 18 years; (2) admission to the Pediatric Oncology or Bone Marrow Transplant (BMT) Unit during the study period; (3) diagnosis of a malignant or hematological disease requiring antineoplastic treatment; (4) documented oral stomatitis and/or perianal mucosal lesions during hospitalization; (5) availability of clinical and nursing documentation regarding lesion localization, treatment approach, treatment initiation, and clinical evolution; (6) availability of sufficient follow-up documentation to determine healing time.

Patients were excluded from the final analytical cohort if the available clinical or nursing documentation was insufficient to reliably determine lesion localization, treatment approach, treatment initiation, clinical evolution, or healing time. When more than one eligible hospitalization was identified for the same patient during the study period, only the first eligible episode was considered.

Oral stomatitis and perianal mucosal lesions were analyzed separately because of their different anatomical localization, clinical assessment, severity classification, and treatment approach.

### 2.3. Statistical Analysis

Data was analyzed using both descriptive and inferential statistical methods. Descriptive statistics were used to summarize demographic, clinical, and nursing variables. Continuous variables were reported as mean and standard deviation (SD), whereas categorical variables were expressed as frequencies and percentages.

The independent samples *t*-test was used to assess whether statistically significant differences existed in the mean values among the treated groups for the selected continuous outcomes. The assumption of normality was assessed using the Shapiro–Wilk test. Continuous variables with an approximately normal distribution were compared using the independent-samples *t* test, whereas the Wilcoxon rank-sum test was used for variables that did not meet the normality assumption.

Exploratory unadjusted analyses were performed to describe the distribution of selected demographic and clinical characteristics across treatment groups. These exploratory analyses were intended to characterize the study population. In addition, separate multivariable linear regression models were performed to evaluate the association between treatment approach and healing time after adjustment for clinically relevant covariates.

Separate multivariable linear regression models were fitted for oral stomatitis and perianal fissures, with healing time (days) as the dependent variable. For oral stomatitis, the model included treatment group, age, WHO oral mucositis grade, antineoplastic treatment modality, and diagnosis group. For perianal fissures, the model included treatment group, age, antineoplastic treatment modality, and diagnosis group, whereas WHO grade was not included because it is specific to oral mucosal toxicity. To avoid model overparameterization, cancer diagnoses were grouped as hematologic or solid malignancies. Regression coefficients were reported with 95% confidence intervals and *p*-values. For comparisons of healing time between treatment groups, Cohen’s d was calculated to quantify the magnitude of the treatment effect, complementing the reporting of mean differences and 95% confidence intervals.

Survival analysis methods were not applied because complete healing was documented for all patients included in the comparative analyses, with no censored observations or losses to follow-up. Accordingly, healing time was analyzed as a continuous outcome using linear regression models.

All statistical tests were two-sided, with a significance level set at α = 0.05. Statistical analyses were performed using IBM SPSS Statistics^®^, version 29.0 (IBM Corp., Armonk, NY, USA).

### 2.4. Ethical Considerations

The study was conducted in accordance with the Declaration of Helsinki and approved by the Pediatric Ethics Committee of the Tuscany Region (Prot. 118/2025; approval date: 13 May 2025). Given the retrospective observational design of the study, the use of pseudonymized data collected during routine clinical practice, and the authorization granted by the Ethics Committee, the requirement for written informed consent was waived in accordance with the applicable national regulations governing scientific research conducted by IRCCS.

## 3. Results

### 3.1. Demographics of the Sample Analyzed

**Figure 1:**
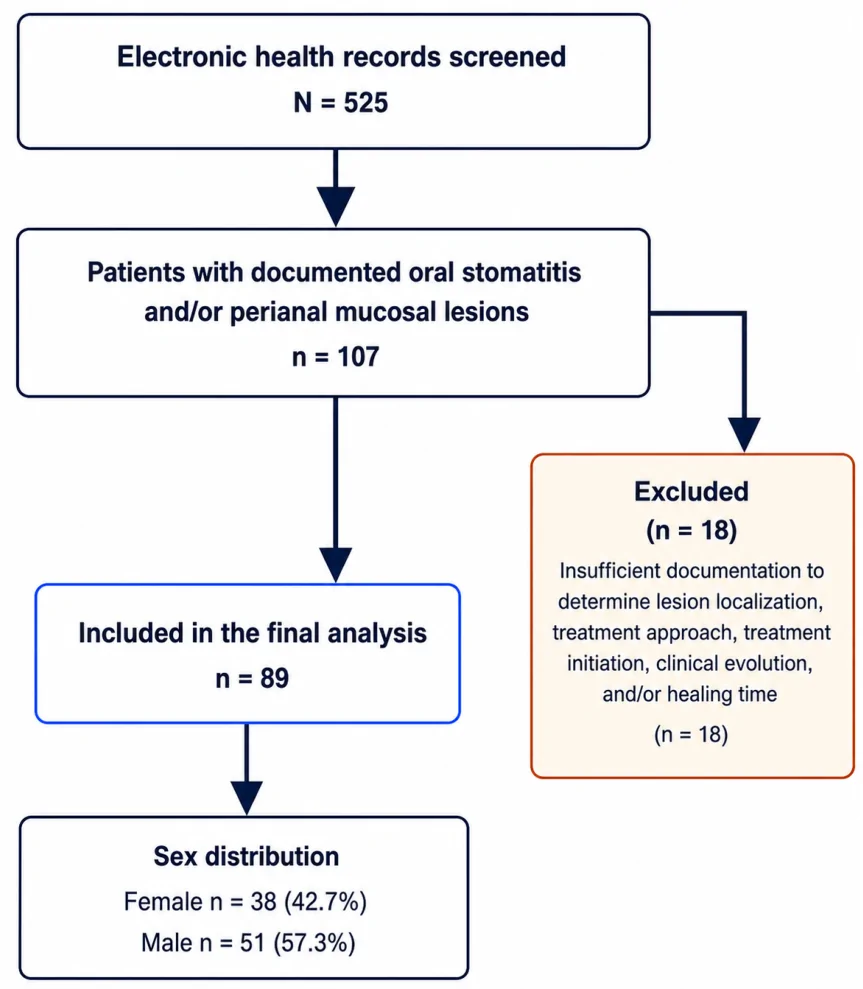
STROBE flow diagram of patient selection.

The research team screened the EHRs of 525 patients admitted to the hospital’s Oncology (n = 478) or BMT Department (n = 47). Among the 107 patients with documented oral stomatitis and/or perianal mucosal lesions, 18 were excluded because of insufficient clinical or nursing documentation, resulting in a final analytical cohort of 89 patients. The patient selection process is summarized in Figure 1. The final sample consisted of 89 patients, including 38 females (42.7%) and 51 males (57.3%), aged between 5 months and 18 years. The demographic characteristics, including cancer diagnosis at admission, are reported in Table 1.

### 3.2. Clinical Data

At the beginning of the observation period, mucosal lesions were already documented in 18.0% of patients, whereas the remaining cases developed after the administration of antineoplastic therapy during hospitalization. Lesions were localized exclusively in the oral cavity in 35 patients (39.3%), exclusively in the perianal region in 38 (42.7%), and in both sites in 16 (18.0%). Among the 51 patients with oral involvement, 19 (37.3%) had grade 2 mucositis, 24 (47.1%) had grade 3, and 8 (15.7%) had grade 4 according to the WHO Oral Toxicity Scale (Table 2). No cases of grade 1 oral mucositis were observed (Table 2).

The antineoplastic treatments administered included chemotherapy alone in 38.2% of patients, radiotherapy alone in 33.7%, and combined chemotherapy and radiotherapy in 28.1%. The antineoplastic agents are reported in Table 2.

Most patients (65.2%) were receiving enteral or parenteral nutritional support, whereas 34.8% maintained oral nutrition (Table 2).

Exploratory unadjusted analyses did not identify statistically significant differences in age (*p* = 0.12), antineoplastic therapy (*p* = 0.56), lesion localization (*p* = 0.52), or nutritional support (*p* = 0.78) between treatment groups. These analyses were descriptive and were not intended to establish baseline equivalence or exclude residual confounding.

Conventional therapy consisted of honey alone in 14.6% of patients and vitamin E spray-based regimens in 32.6% (Table 2). Adjunctive treatments associated with vitamin E spray included 0.2% chlorhexidine (11.2%), nystatin (12.4%), and combinations of honey with chlorhexidine and/or nystatin (9.0%). Treatment distribution according to lesion localization site is reported in Table 3.

As some patients received more than one topical treatment, percentages are not mutually exclusive and may exceed 100%. Treatment groups were therefore classified according to the principal therapeutic approach recorded in the medical chart.

### 3.3. Association Between Treatment Approach and Healing Time

The study examined the treatment approaches used for oral mucositis and their relationship with healing time. Healing time differed significantly between patients treated with conventional therapy and those treated with two class IIb medical devices, namely Mucosamin^®^ Spray and Mucosamin^®^ Rectal Gel (Professional Dietetics S.p.A., Milan, Italy). Overall, Mucosamin^®^-based therapy was used in 52.8% of patients, while the remaining patients received conventional treatment. Therefore, subsequent analyses focused on comparing healing time between these two treatment approaches. For the perianal lesion analysis, the Rectal Gel-containing treatment group included patients treated with Mucosamin^®^ Rectal Gel alone or in combination with Mucosamin^®^ Spray. The conventional treatment group included patients receiving the predefined conventional topical regimens for perianal lesions. The spray was used for oral mucositis, whereas the gel was used for perianal fissures. Conventional treatment consisted of honey alone in 14.6% of patients and combinations of vitamin E spray with chlorhexidine, nystatin, or honey in 32.6% (Table 2).

Baseline demographic and clinical characteristics are presented in Table 4. Panel A summarizes the oral stomatitis cohort, whereas Panel B summarizes the perianal fissure cohort. No statistically significant differences were observed between treatment groups in either analysis with respect to the main demographic and clinical variables, including age, sex, diagnosis, and antineoplastic treatment modality. Baseline WHO oral mucositis grade was also comparable between treatment groups in the oral stomatitis cohort.

**Figure 2:**
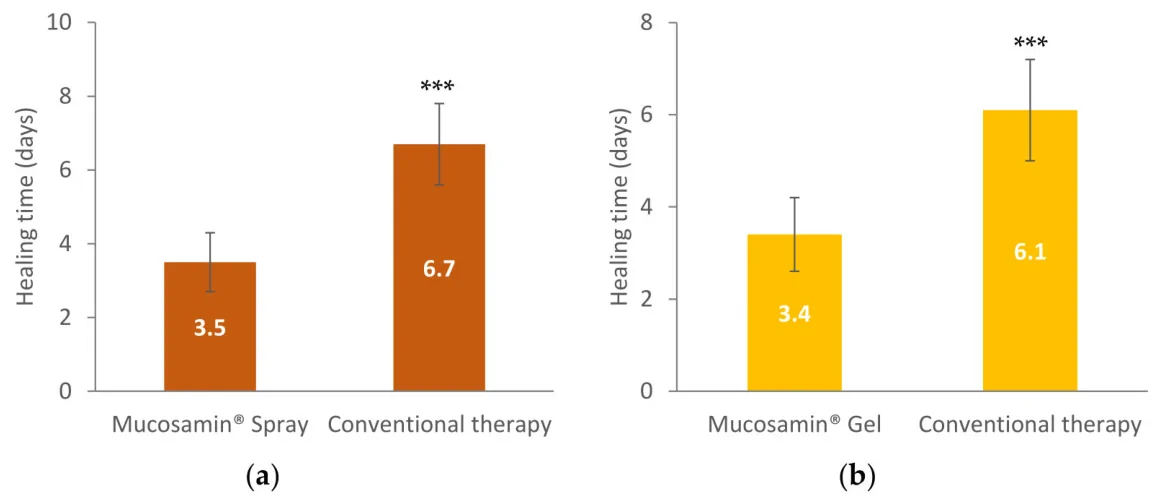
Healing time according to treatment approach. (**a**) Oral stomatitis treated with Mucosamin^®^ Spray versus conventional therapy. (**b**) Perianal fissures treated with Mucosamin^®^ Rectal Gel versus conventional therapy. Data are expressed as mean ± standard deviation. *** *p* < 0.001.

Oral stomatitis represented 57.3% of all cases and was treated with Mucosamin^®^ Spray in 49.0% of patients or with conventional therapy based on vitamin E spray combined with chlorhexidine, nystatin, or honey in 51.0%. Patients treated with Mucosamin^®^ Spray had a mean healing time of 3.5 ± 0.8 days, compared with 6.7 ± 1.1 days in the conventional therapy group, corresponding to an unadjusted mean difference of −3.21 days (95% CI, −3.75 to −2.67; *p* < 0.001) (Figure 2a and Table 4). Mean baseline WHO oral mucositis severity scores were 2.9 ± 0.7 in the Mucosamin^®^ Spray group and 2.7 ± 0.7 in the conventional therapy group. The unadjusted between-group comparison was not statistically significant (*p* = 0.30).

Perianal fissures, either isolated or combined with oral involvement, accounted for 60.7% of all cases. Among patients with perianal lesions included in the comparative analysis, 24 received a treatment approach including Mucosamin^®^ Rectal Gel, whereas 15 received conventional therapy. Patients treated with Mucosamin^®^ Rectal Gel had a mean documented healing time of 3.4 ± 0.8 days, compared with 6.1 ± 0.8 days in the conventional therapy group, corresponding to an unadjusted mean difference of −2.76 days (95% CI, −3.30 to −2.21; *p* < 0.001) (Figure 2b and Table 5).

### 3.4. Multivariable Analysis of Healing Time

In the multivariable analysis of oral stomatitis, treatment with Mucosamin^®^ Spray remained associated with a shorter healing time after adjustment for age, WHO oral mucositis grade, antineoplastic treatment modality, and diagnosis group. The adjusted mean difference was −3.26 days (95% CI, −3.89 to −2.64; *p* < 0.001). The model had an R^2^ of 0.771 and an adjusted R^2^ of 0.734.

For perianal fissures, treatment approaches including Mucosamin^®^ Rectal Gel also remained associated with a shorter healing time after adjustment for age, antineoplastic treatment modality, and diagnosis group. The adjusted mean difference was −2.76 days (95% CI, −3.31 to −2.21; *p* < 0.001). The model had an R^2^ of 0.779 and an adjusted R^2^ of 0.746. No relevant multicollinearity was identified in either model (Table 6).

## 4. Discussion

Therapy-associated mucosal lesions, including oral stomatitis and perianal fissures, represent clinically relevant complications in pediatric oncohematology patients, with potential consequences for pain, feeding, hydration, infection risk, quality of life, and continuity of oncological treatment. In this retrospective observational study, we evaluated the clinical characteristics of mucositis in pediatric oncological patients and analyzed the relationship between different treatment approaches and healing time. The main finding was that patients treated with Mucosamin^®^ Spray for oral stomatitis or Mucosamin^®^ Rectal Gel for perianal fissures showed shorter documented healing times than those receiving conventional therapeutic approaches. In our cohort, most lesions were documented during hospitalization after exposure to chemotherapy, radiotherapy, or combined antineoplastic treatments. This finding confirms the high vulnerability of pediatric patients undergoing chemotherapy, radiotherapy, or combined antineoplastic treatments to mucosal injury. The high occurrence of mucositis observed after therapy is consistent with previous evidence indicating that pediatric patients are at particularly high risk of developing mucosal toxicity.

Most cases in this study were classified as grade 2 to grade 4 according to the WHO Oral Toxicity Scale, indicating that a substantial proportion of patients experienced clinically relevant mucosal involvement. Moderate to severe oral mucositis can interfere with oral intake and may require nutritional support, analgesic therapy, antimicrobial treatment, and prolonged nursing care. In the present sample, most patients were receiving enteral or parenteral nutritional support, further confirming the clinical burden of mucositis and its potential impact on feeding and hydration. This finding is consistent with the known consequences of mucositis, including dysphagia, odynophagia, reduced nutrient intake, dehydration, weight loss, and the need for replacement or supplementation of oral feeding.

An important aspect of the present study is the evaluation of lesion localization. Mucositis was confined to the oral cavity in 39.3% of patients, involved the perianal region in 42.7%, and affected both areas in 18.0% of patients. These data highlight that mucosal toxicity in pediatric oncology may extend beyond the oral cavity and involve different regions of the gastrointestinal tract. Although oral mucositis is often the primary focus in supportive care, gastrointestinal and perianal manifestations may also cause significant discomfort, pain during evacuation, risk of local irritation, and additional nursing care needs.

The comparison between treatment approaches showed a significant reduction in healing time among patients treated with Mucosamin^®^ medical devices compared with those receiving conventional therapy. Among patients with perianal fissures, the mean documented healing time was 3.4 ± 0.8 days in the Mucosamin^®^ Rectal Gel group and 6.1 ± 0.9 days in the conventional treatment group, corresponding to an unadjusted mean difference of 2.7 days (*p* < 0.001). Similarly, among patients with oral mucositis, Mucosamin^®^ Spray was associated with a mean healing time of 3.5 ± 0.8 days, compared with 6.7 ± 1.1 days in the conventional therapy group, corresponding to a mean difference of 3.2 days. These findings suggest that Mucosamin^®^ Spray and Mucosamin^®^ Rectal Gel may represent useful therapeutic options for supporting mucosal healing in pediatric patients with therapy-associated mucositis.

The potential clinical rationale for these medical devices may relate to their hydrating and mucosal-protective action, which may contribute to maintaining a favorable local microenvironment supportive of skin tissue repair [26]. However, the present study was not designed to investigate mechanisms of action, and these hypotheses should therefore be interpreted as biologically plausible explanations rather than demonstrated causal pathways.

The observed reduction in healing time is clinically relevant. A shorter documented healing time may be clinically relevant in pediatric oncology care, where mucosal pain, feeding difficulties, oral hygiene impairment, and infection risk can rapidly affect the child’s overall condition. Nevertheless, the present study did not directly measure pain, nutritional recovery, infection rates, quality of life, or treatment delays; therefore, these potential implications should be considered hypothesis-generating. In addition, rapid mucosal recovery may be particularly valuable in patients undergoing repeated chemo- therapy cycles, in whom persistent or recurrent mucosal injury may interfere with treatment schedules and supportive-care planning.

The conventional therapies used in this study included honey alone and combinations of vitamin E spray with chlorhexidine, nystatin, or honey. These approaches reflect common supportive-care strategies aimed at reducing irritation, controlling microbial complications, and supporting mucosal repair. However, the significantly longer healing time observed in the conventional therapy group suggests that additional or alternative mucosal-protective approaches may be beneficial. It is important to emphasize that the present study cannot establish therapeutic superiority or causal efficacy, because treatment allocation was not randomized and may have been influenced by lesion severity, localization, clinician preference, product availability, patient tolerance, or other unmeasured clinical factors. Although baseline oral mucositis severity was available descriptively for patients with oral lesions, no multivariable adjustment was performed because of the retrospective design and the limited sample size. Therefore, it cannot be excluded that patients receiving different treatments differed in initial clinical severity, which may have influenced healing time independently of the treatment administered.

Because data were extracted retrospectively from electronic health records, healing time depended on the timing and completeness of clinical and nursing documentation. Variability in documentation practices may have influenced the apparent date of lesion improvement or resolution. Nevertheless, the magnitude of the observed difference in healing time and the statistical significance of the comparison support the need for further prospective investigation.

The present study has some strengths that should be considered alongside its limitations. First, it provides data from a specialized pediatric oncohematology setting, where mucositis is a frequent and clinically important complication. Second, it reflects real-world clinical and nursing practice, including both oral and perianal manifestations of mucosal damage. Third, the study evaluated healing time as a clinically meaningful outcome, directly relevant to patients, caregivers, and healthcare professionals. Finally, the analysis included both conventional treatments and class IIb medical devices, allowing comparison between different therapeutic strategies used in routine care. However, several limitations must be acknowledged. The retrospective observational design limits the ability to establish causal relationships between treatment and healing outcomes. Treatment choice may have been influenced by clinical judgment, lesion severity, localization, patient condition, treatment availability, or other factors not fully captured in the electronic health records. Patients were classified according to the principal topical treatment documented in the medical record, and the conventional treatment groups represented predefined alternative therapeutic regimens rather than additional concomitant topical therapies. Nevertheless, because treatment allocation reflected routine clinical practice, was not randomized, and no multivariable adjustment was performed, residual confounding cannot be excluded. Therefore, the observed differences should be interpreted as unadjusted associations rather than evidence of treatment effectiveness. Therefore, residual confounding cannot be excluded. The study was conducted in a single center, which may limit the generalizability of the findings to other pediatric oncology settings. In addition, the sample size, although clinically relevant, may not be sufficient to perform extensive subgroup analyses according to cancer type, antineoplastic regimen, mucositis grade, nutritional status, or concomitant medications.

Although the multivariable analyses adjusted for the main available clinical covariates, differences in baseline lesion severity may still have influenced the results. Baseline WHO oral mucositis grade was available for oral lesions and was included in the adjusted analysis, whereas no standardized severity measure was routinely recorded for perianal fissures. Therefore, the possibility of residual confounding related to baseline lesion severity should be considered when interpreting the findings.

Healing time was determined from routine clinical and nursing documentation rather than from standardized perspective assessments. Although complete healing was identified according to the first documented record of lesion resolution, some variability in the timing of documentation between clinicians cannot be excluded.

Because all eligible patients for whom complete documentation was available were included, selection bias related to parental consent was likely limited. However, exclusion of patients with incomplete clinical documentation may have introduced information bias.

Despite these limitations, the present study provides useful preliminary evidence suggesting that Mucosamin^®^ spray and Mucosamin^®^ Rectal Gel may be associated with faster healing of mucosal lesions in pediatric patients undergoing antineoplastic therapy.

## 5. Conclusions

In conclusion, this retrospective observational study highlights the high clinical burden of mucositis in pediatric oncology patients and underscores the importance of timely and effective supportive care. Treatment with Mucosamin^®^ Spray for oral stomatitis and Mucosamin^®^ Rectal Gel for perianal fissures was associated with shorter documented healing times compared with conventional therapy. These findings reflect associations observed in routine clinical practice and should not be interpreted as evidence of a causal treatment effect. Prospective, adequately powered studies with standardized severity assessment, predefined healing criteria, and appropriate adjustment for potential confounders are needed to confirm these findings and to define standardized, age-appropriate treatment protocols for this vulnerable population.

## Figures and Tables

**Table 1:** Demographic characteristics. Continuous data are expressed as mean ± standard deviation (SD); categorical data are expressed as counts and percentages.

Variables	Total Samples (n = 89)	Units
Age	106.7 ± 61.8	Months
Sex		
Female	42.7% (n = 38)	% (no.)
Male	57.3% (n = 51)	% (no.)
Weight	31.7 ± 16.2	kg
Cancer diagnosis		
Hodgkin lymphoma	23.6% (n = 21)	% (no.)
Acute lymphoblastic leukemia	22.5% (n = 20)	% (no.)
Acute myeloid leukemia	15.7% (n = 14)	% (no.)
Glioblastoma	11.2% (n = 10)	% (no.)
Neuroblastoma	7.9% (n = 7)	% (no.)
Wilms tumor (nephroblastoma)	4.5% (n = 4)	% (no.)
Medulloblastoma	3.4% (n = 3)	% (no.)
Osteosarcoma	3.4% (n = 3)	% (no.)
Retinoblastoma	3.4% (n = 3)	% (no.)
Ewing sarcoma	2.2% (n = 2)	% (no.)
non-Hodgkin lymphoma	2.2% (n = 2)	% (no.)

**Table 2:** Clinical data. Data are expressed as counts and percentages.

Variables	N	%
Lesion localization (n = 89)		
Oral stomatitis	35	39.3
Perianal fissures	38	42.7
Oral stomatitis and perianal fissures	16	18.0
Oral mucositis severity according to the WHO Oral Toxicity Scale * (n = 51)		
Grade 0 (No oral mucositis)	38	42.7
Grade 1 (Erythema and soreness)	0	0.0
Grade 2 (Ulcers, able to eat solids)	19	37.3
Grade 3 (Ulcers, requires a liquid diet ^1^)	24	47.1
Grade 4 (Ulcers, alimentation not possible ^1^)	8	15.7
Pharmacological treatments		
Mucosamin^®^ Spray	31	34.8
Mucosamin^®^ Rectal Gel	16	18.0
Honey	13	14.6
Vitamin E spray + 0.2% chlorhexidine	10	11.2
Vitamin E spray + nystatin	11	12.4
Vitamin E spray + 0.2% chlorhexidine + honey	4	4.5
Vitamin E spray + nystatin + honey	4	4.5

^1^ Due to mucositis. * WHO Oral Toxicity Scale scores were calculated only for patients with documented oral stomatitis (n = 51). Patients with isolated perianal lesions were not included in the oral severity classification.

**Table 3:** Treatment distribution according to lesion localization site. Data are expressed as counts and percentages.

Treatment		Oral Mucositis	Perianal Fissures	Oral and Anal	Total
Mucosamin^®^ Spray	N	14	5	0	19
	%	40.0	13.2	0.0	21.3
Mucosamin^®^ Rectal Gel	N	0	16	0	16
	%	0.0	42.1	0.0	18.0
Mucosamin^®^ Spray + Gel	N	4	1	7	12
	%	11.4	2.6	43.8	13.5
Honey	N	0	13	0	13
	%	0.0	34.2	0.0	14.6
Vitamin E spray + 0.2% chlorhexidine	N	6	1	3	10
	%	17.1	2.6	18.8	11.2
Vitamin E spray + nystatin	N	5	2	4	11
	%	14.3	5.3	25.0	12.4
Vitamin E spray + 0.2% chlorhexidine + honey	N	2	0	2	4
	%	5.7	0.0	12.5	4.5
Vitamin E spray + nystatin + honey	N	4	0	0	4
	%	11.4	0.0	0.0	4.5
Total	N	35	38	16	89
	%	100.0	100.0	100.0	100.0

**Table 4:** Baseline demographic and clinical characteristics according to treatment group. Panel (**A**) reports the comparison between treatment groups in patients with oral stomatitis. Panel (**B**) reports the comparison between treatment groups in patients with perianal fissures.

(**A**) Oral Stomatitis			
**Characteristic**	**Mucosamin^®^** **Spray** **(n = 25)**	**Conventional Therapy** **(n = 26)**	***p*** **Value**
Age, months, mean ± SD	98.8 ± 59.4	111.8 ± 59.4	0.440
Male sex, n (%)	15 (60.0)	16 (61.5)	1.000
WHO oral mucositis grade, mean ± SD	2.88 ± 0.67	2.69 ± 0.74	0.344
Diagnosis, n (%)			1.000
Hematologic malignancy	19 (76.0)	20 (76.9)	
Solid malignancy	6 (24.0)	6 (23.1)	
Antineoplastic treatment modality, n (%)			0.443
Chemotherapy	11 (44.0)	7 (26.9)	
Radiotherapy	8 (32.0)	11 (42.3)	
Combined therapy	6 (24.0)	8 (30.8)	
(**B**) Perianal Fissures			
**Characteristic**	**Mucosamin^®^ Rectal Gel-Based** **Treatment (n = 24)**	**Conventional Therapy** **(n = 15)**	***p*** **Value**
Age, months, mean ± SD	112.3 ± 66.4	102.9 ± 66.8	0.671
Male sex, n (%)	12 (50.0)	7 (46.7)	1.000
Diagnosis, n (%)			0.453
Hematologic malignancy	17 (70.8)	13 (86.7)	
Solid malignancy	7 (29.2)	2 (13.3)	
Antineoplastic treatment modality, n (%)			0.782
Chemotherapy	8 (33.3)	6 (40.0)	
Radiotherapy	9 (37.5)	4 (26.7)	
Combined therapy	7 (29.2)	5 (33.3)	

Data are presented as mean ± standard deviation or n (%), as appropriate. Continuous variables were compared using the independent-samples *t*-test. Categorical variables were compared using Pearson’s χ^2^ test or Fisher’s exact test, as appropriate.

**Table 5:** Comparison of healing time between treatment groups according to lesion localization.

Lesion	Treatment Group	n	Healing Time (Days), Mean ± SD	Mean Difference (Days), 95% CI	*p* Value	Cohen’s d
Oral stomatitis	Mucosamin^®^ Spray	25	3.52 ± 0.82	−3.21 (−3.75 to −2.67)	<0.001	3.34
	Conventional therapy	26	6.73 ± 1.08			
Perianal fissures	Mucosamin^®^ Rectal Gel-based treatment ^1^	24	3.38 ± 0.77	−2.76 (−3.30 to −2.21)	<0.001	3.47
	Conventional therapy	15	6.13 ± 0.83			

^1^ Includes patients treated with Mucosamin^®^ Rectal Gel alone or in combination with Mucosamin^®^ Spray. Note: Mean differences were calculated as Mucosamin^®^-based treatment minus conventional therapy; therefore, negative values indicate shorter healing times in the Mucosamin^®^ groups. Ninety-five percent confidence intervals were estimated using Welch’s independent-samples *t*-test. Cohen’s d is reported as the absolute standardized effect size for each between-group comparison.

**Table 6:** Multivariable linear regression analysis of healing time according to lesion localization.

Outcome (Healing Time)	Main Exposure	Adjusted Mean Difference (Days)	95% CI	*p* Value	R^2^	Adjusted R^2^
Oral stomatitis	Mucosamin^®^ Spray vs. conventional therapy	−3.26	−3.89 to −2.64	<0.001	0.771	0.734
Perianal fissure	Mucosamin^®^ Rectal Gel-based treatment vs. conventional therapy	−2.76	−3.31 to −2.21	<0.001	0.779	0.746

Note: The oral stomatitis model was adjusted for age, WHO oral mucositis grade, antineoplastic treatment modality, and diagnosis group. The perianal fissure model was adjusted for age, antineoplastic treatment modality, and diagnosis group. Diagnoses were categorized as hematologic or solid malignancies. Negative coefficients indicate shorter healing times in patients receiving Mucosamin^®^-based treatment. Heteroscedasticity-robust HC3 standard errors were used.

## Data Availability

The raw data supporting the conclusions of this article will be made available by the authors on request.

## Abbreviations

The following abbreviations are used in this manuscript:

CH	Chemotherapy
RT	Radiotherapy
EHR	Electronic Health Records
WHO	World Health Organization
BMT	Bone Marrow Transplant
